# Nutritional and Metabolic Factors, Ethanol and Cholesterol, Interact With Calcium-Dependent *N*-Methyl-D-Aspartate Receptor Inhibition by Tricyclic Antidepressants

**DOI:** 10.3389/fncel.2022.946426

**Published:** 2022-07-04

**Authors:** Sergei I. Boikov, Dmitry A. Sibarov, Sergei M. Antonov

**Affiliations:** I. M. Sechenov Institute of Evolutionary Physiology and Biochemistry, Russian Academy of Sciences, Saint Petersburg, Russia

**Keywords:** NMDA receptors, tricyclic antidepressants, amitriptyline, desipramine, clomipramine, ethanol, cholesterol, calcium

## Abstract

It is known that overexpression of *N*-methyl-D-aspartate receptors (NMDARs) contributes to central sensitization and development of neuropathic pain. Tricyclic antidepressants (TCAs), amitriptyline (ATL), and desipramine (DES) exhibit analgetic anti-NMDAR activity and are commonly utilized for pain therapy. This property is determined by their ability to enhance the calcium-dependent desensitization (CDD) of NMDARs. Coincidently ethanol and cholesterol, the ubiquitous food supplements, also modulate NMDAR CDD. The convergence of the effects of these compounds on a similar calcium-dependent process allows to assume their interaction on NMDARs. Since there is no information on whether ethanol supplementation and cholesterol deficit interfere with TCA inhibition of NMDARs at a cellular level, here we investigated this issue. Whole-cell NMDA-activated currents were recorded in rat cortical neurons of primary cultures to study how the IC_50_ values for TCA inhibition of NMDARs are influenced by ethanol and cholesterol extraction from the plasma membrane with methyl-β-cyclodextrin. Ethanol at 0.03% did not reliably affect the steady-state NMDA-activated currents. At this threshold concentration ethanol, however, increased IC_50_s for ATL and DES abolishing their calcium-dependent inhibition of NMDARs but did not change IC_50_ for clomipramine (CLO), which is calcium-independent. Whereas the ethanol effects on ATL-induced NMDAR inhibition reached a maximum at 2 mM external [Ca^2+^], for DES the maximum was achieved already at 1 mM external [Ca^2+^], that correlates with the manifestation of the calcium-dependent inhibition of NMDARs by these agents. Cholesterol depletion also increased IC_50_s for both ATL and DES abolishing the calcium-dependent inhibition of NMDARs. The restitution of cholesterol in the plasma membrane reversed the ATL IC_50_ back to the low values, by a restoration of calcium-dependence of ATL. These observations are consistent with the explanation that either 0.03% ethanol or cholesterol extraction may interrupt some intermediate step of CDD transduction or augment NMDAR CDD to the maximal level so that ATL and DES could not further enhance CDD. It is likely that anti-NMDAR action of ATL and DES against neuropathic pain could demonstrate peculiarities in therapeutic profiles during cholesterol decline in aging or medical treatments and ethanol supplementations even in quantities that are insufficient to cause the symptoms of intoxication.

## Introduction

The unique physiological value of *N*-methyl-D-aspartate receptors (NMDARs) in the CNS is generally based on numerous types of their functional regulations (review Traynelis et al., 2010). In particular, NMDARs play a crucial role in central sensitization and development of neuropathic pain after traumatic nerve injury (Zhou et al., 2011; Chen et al., 2014). Clearly, nerve injury promotes neuropathic pain by excessive NMDAR expression in the CNS and the dorsal root ganglia (Chen et al., 2018) which enhances NMDAR activity at both pre- and postsynaptic sites (Chen et al., 2014). This is the reason why NMDAR antagonists are considered to be utilized for a reduction of hyperalgesia in animal models (for review Zhou et al., 2011; Belinskaia et al., 2019). Tricyclic antidepressants (TCAs), amitriptyline (ATL), and desipramine (DES) are widely used for this therapy and exhibit anti-NMDAR properties. Indeed ATL and DES effects against neuropathic pain depend on NMDARs (Eisenach and Gebhart, 1995), rather than a modulation of monoamine uptake (Gillman, 2007).

The important property of NMDARs is, also, the Ca^2+^-dependent desensitization (CDD), which manifests a decrease of macroscopic currents, despite the continuous presence of the agonists (Zhang et al., 1998; Sessoms-Sikes et al., 2005). The Ca^2+^ ions entering through the activated NMDAR channels form a Ca^2+^-calmodulin complex, which triggers CDD by the competitive displacement of α-actin from intracellular Ca^2+^-calmodulin binding domains of NMDAR GluN1 subunits (Legendre et al., 1993; Vyklicky, 1993; Ehlers et al., 1996; Zhang et al., 1998). NMDAR CDD can also be induced by Ca^2+^ entry via neighboring NMDARs (Iacobucci and Popescu, 2019) as well as Ca^2+^ permeable AMPA receptors (Kyrozis et al., 1995) and voltage-gated Ca^2+^ channels (Legendre et al., 1993). The Na^+^/Ca^2+^-exchangers co-localized with NMDARs within the lipid rafts probably share the common near-membrane Ca^2+^-microdomain, representing a Ca^2+^- regulatory unit in close proximity of the inner membrane surface (Marques-da-Silva and Gutierrez-Merino, 2012, 2014). These facts may, presumably, explain why the inhibition of Na^+^/Ca^2+^-exchangers with KB-R7943 (Sibarov et al., 2015) or lithium (Sibarov et al., 2018) promotes NMDAR CDD. Observations, that binding of intracellular Ca^2+^ with BAPTA (Iacobucci and Popescu, 2020) prevents NMDAR CDD and abolishes the KB-R7943 effects on NMDARs (Sibarov et al., 2015) favor this conclusion.

Amitriptyline and desipramine possess the property of comorbid inhibition of NMDAR currents (Stepanenko et al., 2022). Two modes of ATL and DES action on NMDARs are demonstrated, which could be characterized as the open-channel block (Reynolds and Miller, 1988; Sernagor et al., 1989; Barygin et al., 2017) and the Ca^2+^-dependent inhibition (Stepanenko et al., 2019, 2022). Only the latter mechanism, however, can contribute to the therapy, because at TCA concentrations below 1 μM found in the blood plasma of patients (Santos et al., 2017) the open-channel block of NMDARs by ATL (Stepanenko et al., 2019) or DES (Stepanenko et al., 2022) is negligible. It is suggested that the Ca^2+^-dependence of ATL and DES action originates from their ability to enhance the NMDAR CDD (Stepanenko et al., 2019, 2022).

Coincidently ethanol inhibition of NMDARs also requires intracellular calcium accumulation at the close proximity of calmodulin binding intracellular domain of NMDARs (Krupp et al., 1999; Anders et al., 2000). NMDARs of GluN1/2A subunit composition are more sensitive to ethanol inhibition than GluN1/2C receptors (Mirshahi et al., 1998). This correlates with their ability to undergo CDD which is more pronounced for GluN2A-containing receptors (Krupp et al., 1996). In the cerebral cortex expressing di- and triheteromeric NMDARs containing GluN1, GluN2A, and GluN2B subunits (Sheng et al., 1994; Li et al., 1998) the Ca^2+^-dependent inhibition of NMDARs by ethanol is characterized by the IC_50_ value of 20 mM (0.1%) at 1–2 mM extracellular calcium (Boikov et al., 2020). Loading neurons with BAPTA abolished the Ca^2+^-dependent inhibition of NMDARs by ethanol (Boikov et al., 2020).

Another metabolic factor, cholesterol, maintains the integrity of plasma membrane lipid microdomains allowing clustering of NMDARs, Ca^2+^-channels, and transporters (Marques-da-Silva and Gutierrez-Merino, 2012, 2014). Cholesterol depletion by statins reduces the association of NMDARs to lipid rafts (Ponce et al., 2008) and increases either of their Ca^2+^-independent (Korinek et al., 2015) and -dependent (Boikov et al., 2020) desensitizations. Cholesterol extraction from the plasma membrane with methyl-β-cyclodextrin (MβCD) affects the co-localization of NMDARs with Na^+^/Ca^2+^-exchangers (Marques-da-Silva and Gutierrez-Merino, 2014). In experiments on cortical neurons, this procedure enhanced NMDAR CDD and abolished the modulation of NMDAR CDD by the Na^+^/Ca^2+^-exchangers (Sibarov et al., 2015, 2018).

Both ethanol and cholesterol are ubiquitous food supplements and important endogenous metabolic factors. Changes in ethanol and cholesterol tissue contents may originate from the diet as well as from specific therapeutic procedures. Taking into account that both compounds presumably target a common Ca^2+^-dependent process which, in addition, is probably involved in the TCA action on NMDARs, we can hypothesize that ethanol and cholesterol may interfere with TCA inhibition of NMDARs. This aspect of a possible convergence of effects of nutrients and metabolites and medicines on the same molecular target has not been studied yet.

Considering the lack of information on how ethanol and cholesterol may interfere with the TCA action on NMDARs, here we investigate the effects of these compounds on TCA inhibition of currents through native di- and triheteromeric NMDARs composed of GluN1, GluN2A, and GluN2B subunits (Sheng et al., 1994; Zhong et al., 1994; Li et al., 1998) in rat cortical neurons. We demonstrate that 0.03% ethanol, which represents a threshold concentration of NMDAR Ca^2+^-dependent inhibition, and an acute cholesterol extraction from plasma membrane substantially weaken the effects of ATL and DES, but not CLO, on NMDARs. The observations presented here may allow an improvement of TCA therapeutic protocols considering the current states of patients with respect to their diet and metabolism.

## Materials and Methods

### Primary Culture of Cortical Neurons

All procedures using animals were performed according to the guidelines of the Federation for Laboratory Animal Science Associations (FELASA) and were approved by the Animal Care and Use Committees of I. M. Sechenov Institute of Evolutionary Physiology and Biochemistry. The 17-days pregnant rats were provided by I. M. Sechenov Institute of Evolutionary Physiology and Biochemistry Animal Facility. Animals were sacrificed by 1 min CO_2_ inhalation in a plastic container connected to a CO_2_ tank. Fetal brains were used to obtain primary cultures of rat cortical neurons using conventional procedures as described earlier (Mironova et al., 2007). Neurons were incubated in Neurobasal culture medium supplemented with B-27 (Thermo Fisher Scientific, United States) on 7 mm glass coverslips coated with poly-D-lysine. Experiments were performed after 10–14 days in culture (Mironova et al., 2007; Han and Stevens, 2009).

### Patch Clamp Recordings

Whole-cell currents from cultured rat cortical neurons were recorded using a MultiClamp 700B patch-clamp amplifier, low-pass filtered at 400 Hz, and digitized at the acquisition rate of 20 kilosamples per second using Digidata 1440A and pCLAMP v10.6 software (Molecular Devices). Solution exchange was performed by means of a fast solution application system as described earlier (Sibarov et al., 2015). The external bathing solution contained (in mM): 144 NaCl; 2.8 KCl; 1 or 2 CaCl_2_; 10 HEPES, at pH 7.2–7.4, osmolarity 310 mOsm. The pipette solution contained (in mM): 120 CsF, 10 CsCl, 10 EGTA, and 10 HEPES, osmolarity 300 mOsm, with pH adjusted to 7.4 with CsOH. Patch pipettes of 4–6 MΩ were pulled from Sutter BF150-89-10 borosilicate glass capillaries. Experiments were performed at room temperature (22–25°C). Currents were recorded on neurons voltage clamped at −70 mV. Data are reported without corrections for liquid junction potential, which was −11 mV in our experiments. NMDAR currents were elicited by 100 μM NMDA co-applied with 30 μM glycine as a co-agonist. Methyl-β-cyclodextrin (MβCD, 1.5 mM) application for 5 min was used to extract plasma membrane cholesterol.

### Drugs

Compounds were acquired from Sigma-Aldrich, St. Louis, MO, United States. Particularly NMDA M3262, Glycine G7126, Amitriptyline A8404, Desipramine D3900, Clomipramine C7291, Methyl-β-cyclodextrin C4555, Water soluble cholesterol C4951.

### Analysis of Membrane Currents

To determine the blocking potency of TCAs, NMDA elicited currents were measured in the absence and presence of different TCA concentrations ([TCA]). Amplitudes of currents measured in the presence of blocker (I_*b*_) were normalized to maximal current response in control (I_*c*_). The IC_50_ as the concentration of TCA that causes 50% inhibition of currents, and the Hill coefficient (*h*) were estimated by fitting concentration-inhibition curves with the Hill equation: I_*b*_/I_*c*_ = 1/(1 + [TCA]*^h^*/IC_50_*^h^*).

### Statistical Analysis

Data are shown as representative measurements and mean values ± standard error of the mean (SEM). (*n*) refers to the number of recorded cells. Data pairs were compared using an unpaired Student’s two-tailed *t*-test. Multiple groups were compared using one-way or two-way ANOVA and post *hoc* Tukey’s test. Statistical significance is reported in the figures according to the following symbols ^**^, ^***^, and ^****^, which indicate *p*-values below (<) 0.01, 0.001 and 0.0001, respectively. In addition, *p*-values for each comparison are indicated. Curve fitting was performed using OriginPro software (OriginLab Corp.). IC_50_ values obtained from individual experiments performed under the same experimental conditions were averaged to get mean ± SEM values.

## Results

### Tricyclic Antidepressant Inhibition of *N*-Methyl-D-Aspartate Receptors Is Affected by Ethanol

**FIGURE 1:**
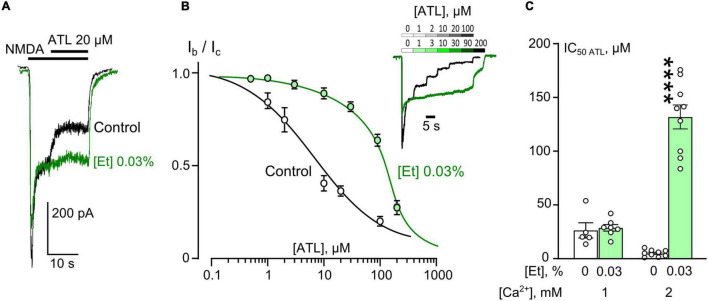
Ethanol effects on amitriptyline (ATL) inhibition of NMDAR currents. **(A)** Currents activated by 100 μM NMDA + 30 μM Gly recorded at –70 mV in a single neuron in the bathing solution containing 2 mM [Ca^2+^] in control and in the presence of 0.03% ethanol. 20 μM ATL was applied at the steady state of NMDA-activated currents. **(B)** Concentration-inhibition curves for ATL of currents activated by 100 μM NMDA + 30 μM Gly in 2 mM [Ca^2+^] in the control bathing solution or in ∼6 mM ethanol ([Et] 0.03%). Symbols show mean values ± SEM of the relative amplitudes of currents (I_b_/I_c_) in the presence (I_b_) and absence (I_c_) of different ATL concentrations ([ATL]). In insert sample traces of NMDAR currents normalized to the steady-state amplitude without ATL, recorded at –70 mV in the presence of increasing [ATL] in control (upper row) and in ethanol (lower row) are shown. **(C)** Dependence of IC_50_ for inhibition of NMDAR currents by ATL on extracellular [Ca^2+^] and ethanol obtained from experiments illustrated in **(A)**. Data from each experiment (symbols) and mean values ± SEM are shown. Mean IC_50_ values are summarized in Table 1. Two-way ANOVA ([Ca^2+^] × [Et]) revealed significant main effects of the [Ca^2+^] [*F*_(1,25)_ = 33.9, *p* = 0.000004] and [Et] [*F*_(1,25)_ = 79.7, *p* = 0.000000003] and interaction between [Ca^2+^] and [Et] [*F*_(1,25)_ = 59.5, *p* = 0.00000005]. Data obtained in 2 mM [Ca^2+^] in ethanol and without ethanol differ significantly (****p = 0.000000003), two-way ANOVA with *post hoc* Tukey’s test.

We, first, started from the observation that the threshold ethanol concentration of 0.03% (6 mM) which itself does not cause any prominent inhibition of NMDAR steady-state currents (Figure 1A), nevertheless abolished the inhibition of NMDAR currents caused by 20 μM ATL. This surprising observation forced us to further study the effects of ethanol on the inhibition of NMDAR currents by three TCAs: ATL, DES, and CLO, which vary with an extent of the Ca^2+^-dependence of their effects on NMDARs (Stepanenko et al., 2019, 2022).

**TABLE 1:** Comparison of IC_50_ values for inhibition by tricyclic antidepressants of NMDAR currents.

		ATL, (μM)	DES, (μM)	CLO, (μM)
[Ca^2+^] 1 mM	Control	19.4 ± 2.2 (*n* = 4) (*h* = 1.6 ± 0.2)	1.75 ± 0.17 (*n* = 15) (*h* = 1.9 ± 0.15)	28 ± 5.6 (*n* = 10) (*h* = 2.9 ± 0.8)
	[Et] 0.03%	28.6 ± 3.0 (*n* = 7) (*h* = 1.6 ± 0.1)	13.6 ± 2.1 (*n* = 10)**** *p* = 0.00000008 (*h* = 1.8 ± 0.2)	49 ± 11 (*n* = 7) (*h* = 2.4 ± 0.2)
[Ca^2+^] 2 mM	Control	5.0 ± 1.0 (*n* = 9) (*h* = 1.2 ± 0.1)	2.2 ± 0.4 (*n* = 11) (*h* = 1.5 ± 0.1)	72 ± 12 (*n* = 12) (*h* = 2.4 ± 0.3)
	[Et] 0.03%	131 ± 33 (*n* = 9)**** p = 0.000000003 (*h* = 1.7 ± 0.18)	10.7 ± 2.0 (*n* = 10)*** *p* = 0.00011 (*h* = 2.0 ± 0.2)	58 ± 9 (*n* = 7) (*h* = 1.7 ± 0.2)
	MβCD	175 ± 36 (*n* = 8)**** p = 0.00004 (*h* = 0.62 ± 0.10)	16.5 ± 4.6 (*n* = 7)** *p* = 0.0082 (*h* = 1.2 ± 0.4)	–
	Cholesterol repletion	20 ± 4.0 (*n* = 10) (*h* = 0.82 ± 0.10)	–	–

**, ***,**** for each tricyclic antidepressant the data are significantly different from control values obtained for corresponding [Ca^2+^]. Data pairs were compared using unpaired two-tailed Student’s t-test. Multiple groups were compared using one-way or two-way ANOVA and post hoc Tukey’s test.

To obtain the concentration-inhibition relationships for ATL effect on NMDARs, ATL concentrations ([ATL]) from 0.5 μM to 200 μM were sequentially applied with an increment to the steady state of NMDA-activated currents (Figure 1B) in 2 mM Ca^2+^-containing bathing solution (2 mM external [Ca^2+^]). The increase of [ATL] induced a progressing decrease of amplitudes of currents. The amplitude values were plotted as a function of [ATL] and the IC_50_ values for ATL inhibition of NMDA-activated currents were measured by fitting the data with the Hill equation. The ATL IC_50_ obtained under control conditions coincides well with the previous data (Stepanenko et al., 2019). In the presence of 0.03% ethanol, however, the concentration-inhibition relationships shifted toward a larger [ATL] so that a 26-fold increase of the ATL IC_50_ value from about 5 μM obtained under control condition to about 131 μM in 0.03% ethanol was observed (Figure 1C and Table 1). It should be noted that in similar experiments in 1 mM external [Ca^2+^] such an increase of the ATL IC_50_ value was not found since these parameters of ATL inhibition of NMDAR currents did not differ in control conditions and in the presence of 0.03% ethanol (Figure 1C and Table 1).

**FIGURE 2:**
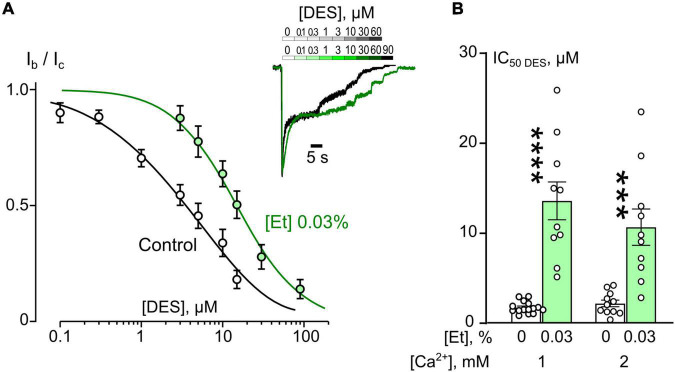
Ethanol effects on desipramine (DES) inhibition of NMDAR currents. **(A)** Concentration-inhibition curves for DES inhibition of currents activated by 100 μM NMDA + 30 μM Gly obtained in 2 mM [Ca^2+^] in the control bathing solution or in ∼6 mM ethanol ([Et] 0.03%). Symbols show mean values ± SEM of the relative amplitudes of currents (I_b_/I_c_) in the presence (I_b_) and absence (I_c_) of different DES concentrations ([DES]). In insert sample traces of NMDAR currents normalized to the steady-state amplitude without DES, recorded at –70 mV in the presence of increasing DES concentrations in control (upper row) and in ethanol (lower row) are shown. **(B)** Dependence of IC_50_ for inhibition of NMDAR currents by DES on extracellular [Ca^2+^] and ethanol obtained from experiments illustrated in **(A)**. Data from each experiment (symbols) and mean values ± SEM are shown. Mean IC_50_ values are summarized in Table 1. Two-way ANOVA ([Ca^2+^] × [Et]) revealed significant main effects of [Et] [*F*_(1,42)_ = 61.7, *p* = 0.0000000009] and no interaction between [Ca^2+^] and [Et]. Data obtained in ethanol and without ethanol differ significantly in both 1 mM [Ca^2+^] (****p = 0.00000008) and 2 mM [Ca^2+^] (***p = 0.00011), two-way ANOVA with *post hoc* Tukey’s test.

In similar experiments, the effects of DES within a wide range of [DES] were studied (Figure 2A). In 1 mM external [Ca^2+^], the DES IC_50_ values of about 1.75 μM and about 13.8 μM were obtained under control conditions and 0.03% ethanol, correspondently. Quite similar DES IC_50_s as in 1 mM external [Ca^2+^] both under control and ethanol were found in 2 mM external [Ca^2+^] suggesting that at these extracellular [Ca^2+^]s ethanol induced a substantial increase of DES IC_50_s by 5–8 folds (Figure 2B and Table 1).

**FIGURE 3:**
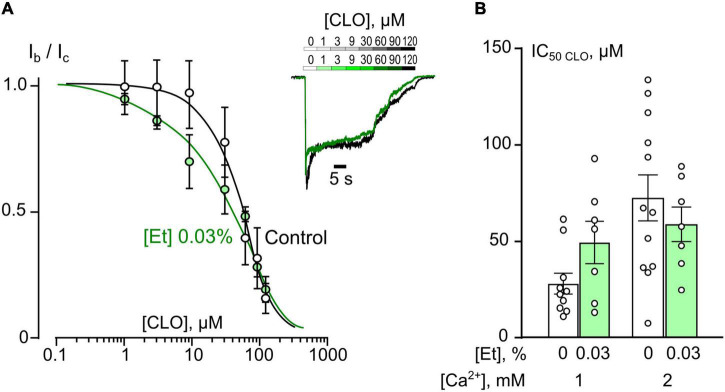
Ethanol do not affect inhibition of NMDAR currents by clomipramine (CLO). **(A)** Concentration-inhibition curves for CLO inhibition of currents activated by 100 μM NMDA + 30 μM Gly obtained in 2 mM [Ca^2+^] in the control bathing solution or in ∼6 mM ethanol ([Et] 0.03%). Symbols show mean values ± SEM of the relative amplitudes of currents (I_b_/I_c_) in the presence (I_b_) and absence (I_c_) of different CLO concentrations ([CLO]). In insert sample traces of NMDAR currents normalized to the steady-state amplitude without CLO, recorded at –70 mV in the presence of increasing CLO concentrations in control (upper row) and in ethanol (lower row) are shown. **(B)** Dependence of IC_50_ for inhibition of NMDAR currents by CLO on extracellular [Ca^2+^] and ethanol obtained from experiments illustrated in **(A)**. Data from each experiment (symbols) and mean values ± SEM are shown. Mean IC_50_ values are summarized in Table 1. Two-way ANOVA ([Ca^2+^] × [Et]) show no significant effects for both [Et] and [Ca^2+^]. Data obtained in ethanol and without ethanol do not differ significantly in both 1 and 2 mM [Ca^2+^].

In accord to the previous observations obtained in the absence of ethanol (Stepanenko et al., 2019, 2022) ATL and DES exhibit a profound Ca^2+^-dependence of NMDAR inhibition suggesting a contribution of CDD in their effects, whereas CLO demonstrates a lack of Ca^2+^-dependence. If ethanol, somehow, may prevent the Ca^2+^-component of ATL and DES inhibition of NMDARs, then the lack of the ethanol effect on the CLO inhibition of NMDARs is expected. To clarify this assumption CLO induced inhibition of NMDAR currents was studied using the same experimental protocol as for ATL and DES (Figure 3A). Ethanol at 0.03% had no effect on CLO IC_50_s of NMDAR inhibition at both 1 mM and 2 mM extracellular [Ca^2+^] (Figure 3B and Table 1). Thus, some correlation between an extent of the Ca^2+^-dependence of TCA inhibition of NMDARs and an extent of ethanol effect on the TCA IC_50_ value appears to exist: if the Ca^2+^-dependence is more pronounced, then the ethanol effect is pronounced too.

### Tricyclic Antidepressant Inhibition of *N*-Methyl-D-Aspartate Receptors Depends on Plasma Membrane Cholesterol

**FIGURE 4:**
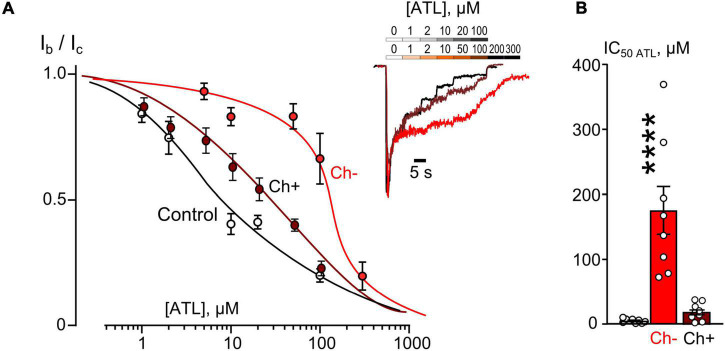
Plasma membrane cholesterol extraction by MβCD affects inhibition of NMDAR currents by amitriptyline (ATL). **(A)** Concentration-inhibition curves for ATL effect on currents activated by 100 μM NMDA + 30 μM Gly obtained in 2 mM [Ca^2+^] in control, after cholesterol extraction with MβCD (Ch−), and after cholesterol repletion with water soluble cholesterol (Ch+). Symbols show mean values ± SEM of the relative amplitudes of currents (I_b_/I_c_) in the presence (I_b_) and absence (I_c_) of different ATL concentrations. In insert sample traces of NMDAR currents normalized to the steady-state amplitude without ATL, recorded at –70 mV in the presence of increasing ATL concentrations in control (upper row) and after cholesterol extraction or repletion (lower row). **(B)** Mean IC_50_s for ATL inhibition of NMDAR currents in control, cholesterol depleted (Ch−) and cholesterol repleted (Ch+) neurons derived from data on **(A)**. Data obtained in cholesterol depleted cells differ significantly from these of control and cholesterol repleted neurons (****p = 0.00004), one-way ANOVA with *post hoc* Tukey’s test. Mean IC_50_ values are summarized in Table 1.

**FIGURE 5:**
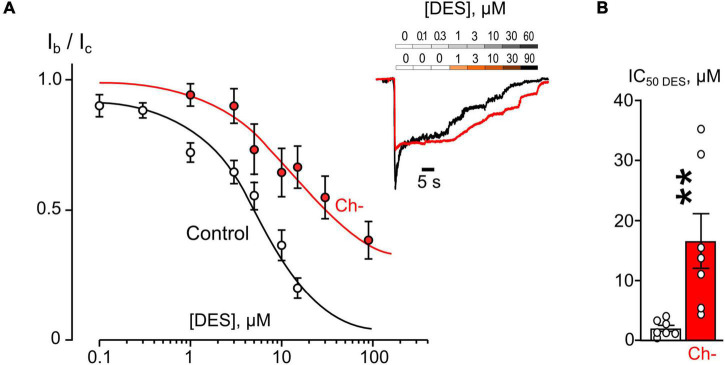
Plasma membrane cholesterol extraction by MβCD affects inhibition of NMDAR currents by desipramine (DES). **(A)** Concentration-inhibition curves for DES effect on currents activated by 100 μM NMDA + 30 μM Gly obtained in 2 mM [Ca^2+^] in control, after cholesterol extraction with MβCD (Ch−). Symbols show mean values ± SEM of the relative amplitudes of currents (I_b_/I_c_) in the presence (I_b_) and absence (I_c_) of different DES concentrations. In insert sample traces of NMDAR currents normalized to the steady-state amplitude without DES, recorded at –70 mV in the presence of increasing DES concentrations in control (upper row) and after MβCD treatment (lower row). **(B)** Mean IC_50_s for DES inhibition of NMDAR currents in control and cholesterol depleted (Ch−) neurons derived from data illustrated on **(A)**. Data obtained in cholesterol depleted cells differ significantly from these of control (**p = 0.0082), unpaired Student’s *t*-test. Data from each experiment (symbols) and mean values ± SEM are shown. The IC_50_ values are summarized in Table 1.

Plasma membrane cholesterol is an important factor of NMDAR CDD modulation (Sibarov et al., 2018). This circumstance poses the question whether the cholesterol deficit may interfere with the Ca^2+^-dependent mode of NMDAR inhibition by TCAs, which presumably is based on an enhancement of NMDAR CDD. To address this point similar experiments, as for ethanol, were performed in which the IC_50_ values for ATL (Figure 4) and DES (Figure 5) inhibition of currents activated by NMDA were measured on neurons after cholesterol extraction with MβCD.

The concentration-inhibition relationships revealed that in 2 mM external [Ca^2+^] the inhibitions by both ATL (Figure 4A) and DES (Figure 5A) of NMDAR currents were affected by cholesterol extraction. In particular, this procedure caused a 35-fold increase of ATL IC_50_ (Figure 4B and Table 1) and a 7-fold increase of DES IC_50_ (Figure 5B and Table 1). We also verified whether the effect of cholesterol extraction on NMDAR inhibition induced by ATL could be reversed. To accomplish this goal, MβCD pre-treated neurons were further incubated for 30 min with 1.5 mM of water-soluble cholesterol to achieve cholesterol repletion into the plasma membrane. The IC_50_ value for ATL inhibition of NMDAR currents in cholesterol repleted neurons was almost 9-folds lesser than the value obtained on neurons after the cholesterol extraction (Figures 4A,B and Table 1). Thus, changes of the cholesterol content in the plasma membrane of neurons induced the reversible effect on the ATL inhibition of NMDAR currents.

The observations presented here exhibit that ethanol and cholesterol, which interfere with NMDAR CDD, may mask and cause an apparent weakening of the Ca^2+^- dependence of ATL and DES effects on NMDARs so that the rest inhibition represents the open-channel block by ATL and DES. In agreement, the pure open-channel blocker of NMDARs, CLO, does not demonstrate any sensitivity to ethanol action.

## Discussion

In general, the convergence of the effects of nutritional and metabolic factors, like ethanol and cholesterol, and TCAs, like ATL and DES, in the most parsimonious way could be explained by the competition for the same molecular targets which causes a considerable weakening of the ATL and DES effects on NMDARs. Because the only known Ca^2+^-dependent process in the kinetics of NMDAR activation is CDD, then the Ca^2+^-dependent NMDAR inhibition by ethanol and the cholesterol extraction from the plasma membranes of neurons and the Ca^2+^-dependent inhibition of NMDARs by ATL and DES are consistent with the interpretation that all these compounds exhibit an enhancement of NMDAR CDD.

### Cholesterol

It has been recently shown that cholesterol depletion increases the rate of NMDAR entry into the desensitized state (Korinek et al., 2015), enhances NMDAR CDD (Sibarov et al., 2018), and is accompanied by a functional uncoupling of NMDAR CDD and Na^+^/Ca^2+^-exchangers (Sibarov et al., 2018). In general, the observation described here, that cholesterol depletion by MβCD treatment drastically increased IC_50_s for ATL and DES inhibition of NMDAR currents by preventing its Ca^2+^-dependent component, is consistent with the above studies. In agreement, the IC_50_ values of TCA-induced NMDAR inhibition on cholesterol deficient cells corresponded well to the NMDAR open-channel block (Stepanenko et al., 2019, 2022). Conversely, the restitution of cholesterol content in the plasma membrane reversed the ATL and DES IC_50_ back to the low values, by a restoration of their Ca^2+^-dependent inhibition. Therefore, our data speak in favor of the conclusion that cholesterol deficit causes the development of NMDAR CDD of the maximal level so that ATL and DES could not add anything to further induction of CDD. This supports our hypothesis that both cholesterol extraction and treatment with ATL or DES may target common Ca^2+^-dependent processes in the chain of the CDD induction.

### Ethanol

The phenomenology of the ethanol action on NMDARs and TCA effects, in general, resembles those of cholesterol. In particular, the high-affinity Ca^2+^-dependent component of ethanol inhibition of NMDARs occurs by CDD enhancement and vanishes when CDD is already fully exacerbated by cholesterol extraction (Boikov et al., 2020). It is, therefore, expected that high-affinity Ca^2+^-dependent inhibition of NMDARs by ethanol at IC_50_ of about 0.1% (Boikov et al., 2020) should abolish the Ca^2+^-dependent TCA inhibition of the receptor. It appeared that even at a threshold concentration of 0.03% at which ethanol does not decrease the steady-state amplitude of NMDA-activated currents, this compound substantially increased the IC_50_ values for NMDAR inhibition by ATL and DES and their magnitudes became similar to the values of the open-channel block (Stepanenko et al., 2019, 2022). Obviously, while used in concentrations that could not induce reliable effects on NMDA-activated currents, ethanol is able to interact with some intermediate steps in the CDD development and to prevent the Ca^2+^-dependent inhibition of NMDARs by ATL and DES. This conclusion and the failure of ethanol to affect the CLO inhibition of NMDARs, both support the enhancement of CDD as the mechanism of ATL and DES Ca^2+^-dependent inhibitions of NMDARs and favor the competition of these TCA and ethanol for similar molecular targets in the chain of CDD induction.

Noticeably, ethanol effects on ATL, DES, and CLO NMDAR inhibition vary substantially with the extracellular [Ca^2+^] changes. In particular, DES inhibition of NMDARs was affected by ethanol when extracellular [Ca^2+^] reached 1 mM, while at least 2 mM Ca^2+^ was required to attenuate the ATL effect. CLO inhibition of NMDARs was not affected by ethanol regardless of [Ca^2+^]. The observed differences between ATL, DES, and CLO correlate with the peculiarities of their NMDARs inhibition (Stepanenko et al., 2019, 2022). Particularly, both the DES Ca^2+^-dependent inhibition of NMDARs and the ethanol effect on this process reach the maximal manifestations at 1 mM of extracellular [Ca^2+^]. The Ca^2+^-dependent inhibition of NMDARs by ATL gains with the extracellular [Ca^2+^] increase to 4 mM (Stepanenko et al., 2019). In parallel, the ethanol effect on the ATL inhibition is weaker at 1 mM, than 2 mM Ca^2+^. As for CLO, this compound blocks NMDA-activated currents in a Ca^2+^-independent fashion (Stepanenko et al., 2022), which, perhaps, represents a background for the lack of the ethanol effect. These observations allow us to suggest an existence of a positive correlation between the manifestation of the Ca^2+^-dependent inhibition of NMDARs by a particular TCA and the susceptibility of this process to ethanol modulation.

It is well established that the Ca^2+^-dependent inhibition of NMDARs by ethanol requires the functional activity of C0 domain of the GluN1 (Anders et al., 2000). Besides this receptor domain, the intracellular chain of participants in the CDD induction involves many molecules including calmodulin, alpha-actin, plasma membrane cholesterol, Ca^2+^ ions, Ca^2+^ transporter, and others (for review Sibarov and Antonov, 2018). Unfortunately, from our experiments, we can not point out directly which particular molecule in this chain is affected by the threshold ethanol concentrations to prevent a function of Ca^2+^ as the intracellular messenger in the CDD enhancement by TCAs. Regardless of the particular protein in the transduction chain of the NMDAR CDD involved in the effects of ATL, DES, ethanol, and cholesterol here we demonstrated possible interference of medicines, nutrients, and metabolic factors that may occur at a cellular level during therapies and complicate therapeutic protocols considering patient diet and metabolic state.

### Conclusion

Based on our observations we can conclude that anti-NMDAR therapeutic effects of ATL and DES against neuropathic pain (Eisenach and Gebhart, 1995; Santos et al., 2017) could be affected by ethanol food supplementation in insufficient quantities to cause the noticeable symptoms of ethanol intoxication. In addition, the Ca^2+^-dependent processes that are involved in ATL and DES NMDAR inhibition also represent targets for cholesterol modulation. Cholesterol, as an important nutrient and metabolite, is subjected to numerous types of modulation in organism tissues, especially in the brain that has the highest content of cholesterol among other organs. However, in some brain regions cholesterol declines with age (Soderberg et al., 1990; Dietschy and Turley, 2004) or due to therapeutic intake of statins (Cibickova, 2011). The type of interaction between drugs, nutrients, and metabolites described here allows us to suggest that cholesterol decline has a complex influence on neuropathic pain in animals and humans (Pergolizzi et al., 2020) and also may affect ATL and DES analgetic properties. This aspect of pharmacological interactions should be considered when assigning the TCA therapy.

## Data Availability Statement

The original contributions presented in this study are included in the article/supplementary material, further inquiries can be directed to the corresponding author/s.

## Ethics Statement

The animal study was reviewed and approved by the Animal Care and Use Committee of I. M. Sechenov Institute of Evolutionary Physiology and Biochemistry.

## Author Contributions

SB: experimental work and data acquisition. DS: data analysis and preparation of figures. SA and DS: study design and interpretation, drafting of the manuscript, and final approval of manuscript. All authors contributed to the article and approved the submitted version.

## Conflict of Interest

The authors declare that the research was conducted in the absence of any commercial or financial relationships that could be construed as a potential conflict of interest.

## Publisher’s Note

All claims expressed in this article are solely those of the authors and do not necessarily represent those of their affiliated organizations, or those of the publisher, the editors and the reviewers. Any product that may be evaluated in this article, or claim that may be made by its manufacturer, is not guaranteed or endorsed by the publisher.
